# Sensitive, Rapid, Quantitative and *in Vitro* Method for the Detection of Biologically Active Staphylococcal Enterotoxin Type E

**DOI:** 10.3390/toxins8050150

**Published:** 2016-05-13

**Authors:** Reuven Rasooly, Paula Do, Bradley Hernlem

**Affiliations:** Western Regional Research Center, Foodborne Toxin Detection & Prevention Research Unit, Agricultural Research Service, United States Department of Agriculture, Albany, CA 94710, USA; paula.do@ars.usda.gov (P.D.); bradley.hernlem@ars.usda.gov (B.H.)

**Keywords:** styphlococcal enterotoxin type E, T-cells, splenocyte

## Abstract

*Staphylococcus aureus* is a major bacterial cause of clinical infections and foodborne illnesses through its production of a group of enterotoxins (SEs) which cause gastroenteritis and also function as superantigens to massively activate T cells. In the present study, we tested Staphylococcal enterotoxin type E (SEE), which was detected in 17 of the 38 suspected staphylococcal food poisoning incidents in a British study and was the causative agent in outbreaks in France, UK and USA. The current method for detection of enterotoxin activity is an *in vivo* monkey or kitten bioassay; however, this expensive procedure has low sensitivity and poor reproducibility, requires many animals, is impractical to test on a large number of samples, and raises ethical concerns with regard to the use of experimental animals. The purpose of this study is to develop rapid sensitive and quantitative bioassays for detection of active SEE. We apply a genetically engineered T cell-line expressing the luciferase reporter gene under the regulation of nuclear factor of activated T-cells response element (NFAT-RE), combined with a Raji B-cell line that presents the SEE-MHC (major histocompatibility complex) class II to the engineered T cell line. Exposure of the above mixed culture to SEE induces differential expression of the luciferase gene and bioluminescence is read out in a dose dependent manner over a 6-log range. The limit of detection of biologically active SEE is 1 fg/mL which is 10^9^ times more sensitive than the monkey and kitten bioassay.

## 1. Introduction

The bacterium *Staphylococcus aureus* is a major cause of clinical infections and foodborne illnesses [1] and produces a range of Staphylococcal Enterotoxins (SEs), twenty-three of which have thus far been identified. Several SEs subtypes have the potential to pose a severe threat to public health and safety and may be produced as an offensive biologic warfare agent [2]. The SEs target the gastrointestinal tract and also behave as superantigens in immune system response. These responses have been shown to be linked [3,4]. Mutation studies demonstrate that the loss of superantigenic activity is correlated with a similar loss in enterotoxicity [5]. SEs bind directly to major histocompatibility complex (MHC) class II of antigen presenting cells (APC) and present them to T-cells [6]. Additionally, without the safety mechanism requiring cellular interaction between APC and T-cells, SEs are able to bind directly to MHC class II expressed on a small subset of naïve CD4^+^ T cells that perform the role of both APC and T cells [7]. This interaction causes massive nonspecific activation of the immune system and stimulates ~20% of the naïve T-cell population [8]. This is 20,000 times higher compared to stimulation with normal antigen and triggers intracellular signals that lead to differential expression of CD4^+^ T cell surface receptor CD154 [9] and substantial secretion of cytokines, such as tumor necrosis factor (TNF) [9], IFN-γ protein [10], and also causes proliferation of T-cells in a time and dose dependent manner [11].

In western Iran the prevalence of Staphylococcal enterotoxin type E (SEE) was the second most frequent subtype and was carried by 18.4% of contaminated food items originating from an animal source [12]. SEE has been detected in *S. aureus* cultures recovered from 17 of the 38 suspected staphylococcal food poisoning incidents [13]. Recent SEE contamination of soft cheese made from unpasteurized milk caused six poisoning outbreaks in France [14]. Additionally, SEE has been associated with three outbreaks in the UK [15]. Outbreaks in the USA, which occurred in Wisconsin, and involved more than 50 people, half of whom were hospitalized [16]. These outbreaks emphasize the need for developing sensitive, quantitative methods to detect active SEE.

Food is routinely heat treated to kill contaminant bacteria and to inactivate their toxins. Thus, it is essential to develop assays that distinguish the biological active form of the toxin, which poses a threat, from the inactive form, which poses no threat. The currently accepted methods for the detection of active SEE are an *in vivo* monkey or kitten bioassay [16,17,18]. Mass spectrometry (MS) and enzyme-linked immunosorbent assays (ELISA) have also been used for SEE detection [17,19] but neither method is able to distinguish active from inactive toxin. Furthermore, antibodies can react with components of the food sample matrix to give falsely positive results [20].

The main objective of this study was to develop rapid sensitive and quantitative bioassays for detection of active SEE. Previously, we showed 6 h after splenocyte stimulation by staphylococcal enterotoxin, the T cell surface receptor CD154 is expressed in a dose dependent manner [21]. It has been shown that the nuclear factor of activated T-cells (NFAT) binds to the promoter region that regulates CD154 protein [22]. Also, it has been shown that the lymphoblastoid B-cell line Raji can perform the role of antigen presenting cell, where SEs are recognized, and these cells interact with human T-lymphocyte Jurkat cells through particular sequences within the variable region of the β chain (V-β8) of the T cell receptor (TCR) [23,24]. In this study, we utilized the mechanism by which superantigens, once presented by MHC II, can activate T cell response. We used Raji cells in combination with a Jurkat T cell-line genetically engineered to express the luciferase reporter gene under the regulation of NFAT response element (NFAT-RE). SEE stimulation in increasing concentrations causes an upregulation of NFAT-RE and bioluminescence in a dose dependent manner.

## 2. Results

### 2.1. Quantitative Splenocyte Proliferation Bioassay for Measuring Biologically Active SEE

**Figure 1:**
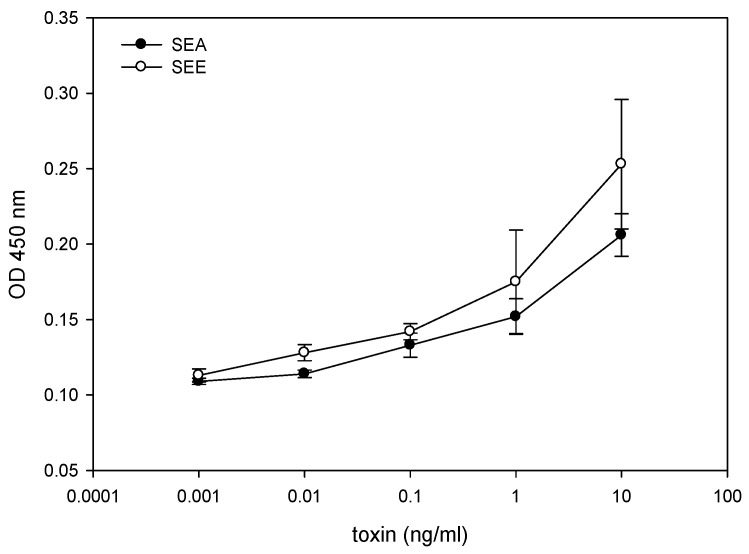
Detection of Staphylococcal enterotoxin type E (SEE) by spleen cell proliferation bioassay. Mouse splenocytes were spiked with increasing concentrations of SEE. After incubation for 2 days, newly synthesized DNA was measured. Error bars represent standard errors and an asterisk indicates significant differences (*p* < 0.05) between spiked and unspiked media.

We evaluated an *ex vitro* assay as an alternative to the monkey and kitten bioassay for measuring biologically active SEE. The splenocyte proliferation assay based on BrdU incorporation was applied to measure the effect of SEE concentrations from 10 ng/mL to 1 pg/mL. The BrdU content of DNA freshly synthesized in proliferating cells was determined by spectroscopic measurement. Figure 1 shows that the amount of newly synthesized DNA measured by BrdU is positively correlated to the concentration of SEE or Staphylococcal enterotoxin type A (SEA). There was a significant difference (*p* < 0.05), in the amount of newly synthesized DNA measured by BrdU incorporation, between cell culture media spiked with SEE or SEA and control unspiked media. The limit of detection was 10 ng/mL of SEE and 1 ng/mL of SEA, while a typical ELISA assay has a detection limit of 1 ng/mL.

### 2.2. Quantitative Bioluminescence—Fast in Vitro Assay for Measuring Biologically Active SEE

**Figure 2:**
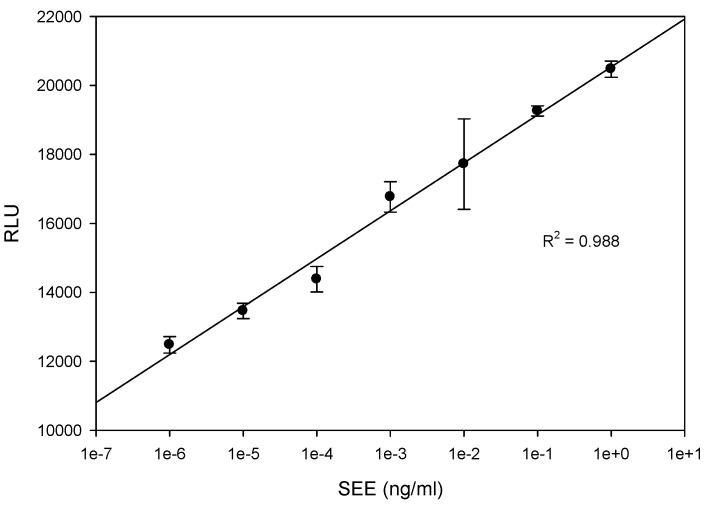
Bioluminescence assay for measuring biologically active SEE. Jurkat reporter cells were plated with Raji cells in a 96-well plate. The mixed cultures were co-incubated with various concentrations of SEE for 5 h. The photon emission was detected by luminometer. Error bars represent standard errors.

To determine whether the luciferase reporter assay can be used for SEE quantification, various concentrations of SEE, ranging from 1 ng/mL to 1 fg/mL, were added to cell mixture containing Jurkat reporter cells and Raji cells. As shown in Figure 2, five hours after exposure to SEE the cells produce high levels of luciferase. The light intensity from the luciferase reaction, the visible outcome of the expression of the luciferase enzyme which catalyzes the luciferin substrate, is proportional to SEE concentration over a 6-log range with linear correlation *R*^2^ = 0.99. In terms of sensitivity, this Jurkat reporter cell line-based assay enables the detection of 1 fg/mL of biologically active SEE, an amount which is 10^6^ times more sensitive than a typical ELISA assay at a detection limit of 1 ng/mL. Although the splenocyte proliferation assay, in Figure 1, showed a proliferation response to both SEE and SEA, SEA exhibited no response in the Jurkat reporter cell line-based assay. This is despite the fact that both SEA and SEE toxins have a segment with the HEXXH zinc-binding motif of metalloproteases [25] and both belong to the same group based on amino acid sequence homology [26,27].

### 2.3. The Effect of Various Pasteurization Time-Temperature Conditions on SEE Activity

**Figure 3:**
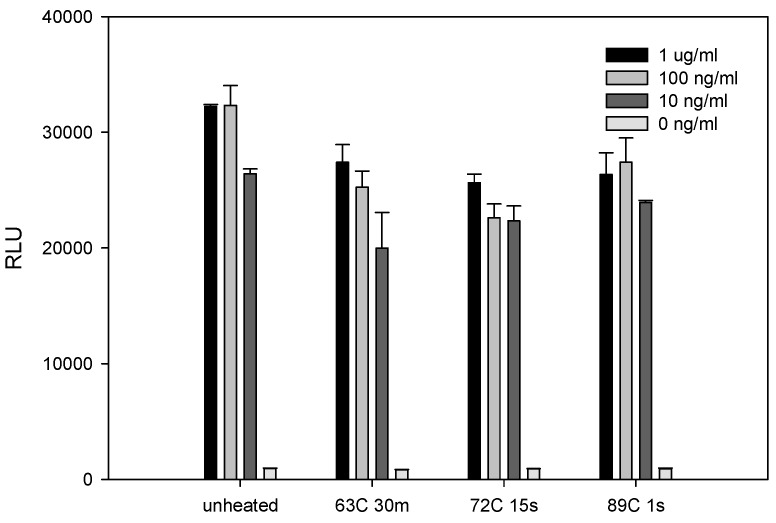
Pasteurization slightly reduces the biological activity of SEE at low concentrations. Phosphate buffered saline (PBS) was spiked with increasing concentrations of SEE and pasteurized at 63 °C for 30 min, 72°C for 15 s, and 89°C for 1 s. Pasteurized, spiked PBS (15 μL) was added to the Jurkat reporter cells. After incubation for 5 h, the luciferase enzyme activity was determined. The photon emission was detected by luminometer. Error bars represent standard errors.

Food is routinely heat treated (pasteurized) to destroy contaminant bacteria. We examined the effect of three different industrial pasteurization conditions for the inactivation of SEE. Various concentrations of SEE in PBS were heat treated and then added to the mixed culture of Raji cells and Jurkat reporter cells. As shown in Figure 3, all of these relatively mild pasteurization conditions slightly reduced SEE toxin activity as measured by decreased luciferase activity. The decrease in activity with pasteurization was most evident at the lowest SEE concentration of 10 ng/mL.

### 2.4. Matrix Effect on Heat Inactivation of SEE

**Figure 4:**
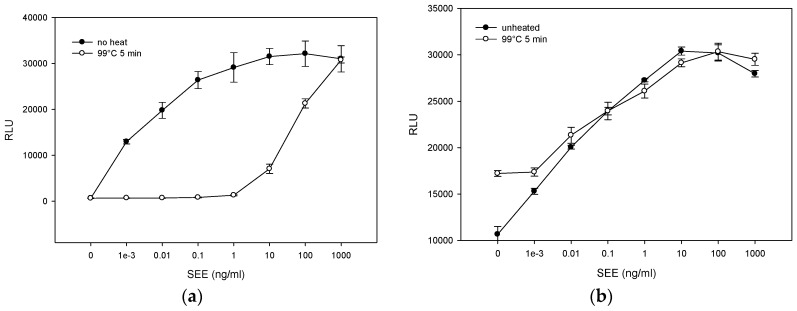
Thermal treatment at 99 °C for 5 min reduces the biological activity of SEE in PBS. However, milk has a protective effect on SEE retained from its initial activity. PBS (**a**) and milk (**b**) spiked with increasing concentrations of SEE were treated at 99 °C for 5 min. Thermal treated spiked PBS or milk (15 μL) was added to the Jurkat reporter cells. After incubation for 5 h, the luciferase enzyme activity was determined. Photon emission was detected by luminometer. Error bars represent standard errors.

To determine whether more severe thermal treatment inactivates SEE, we spiked PBS or milk with various increasing concentrations of SEE ranging from 1 pg/mL to 1 μg/mL and heated the samples at 99 °C for 5 min. As shown in Figure 4a, following heat treatment, there was a sharp decrease in SEE activity in PBS and the reduction in SEE activity was concentration dependent. Lower SEE concentration exhibited a higher decrease in SEE activity. However, milk has a protective effect on SEE. As shown in Figure 4b, SEE in milk was thermostable and retained its initial activity. Furthermore, the results in Figure 4 also show that reconstituted milk induced luciferase background activity. Specifically, without milk the luciferase reporter gene generated low autoluminescence background activity. Milk caused the Relative Light Unit (RLU) to increase by 16 times, from 675 ± 41 RLU (Figure 4a) to 10,655 ± 851 RLU (Figure 4b). Previously, it was shown that reconstituted milk enhances the production of firefly luciferase expressed in transduced mammalian cells [28]. We speculate that milk binds nonspecifically to regulatory sequences and activates the response element controlling luciferase expression. Consequently, the signal to noise ratio decreased and the level of detection in milk was reduced to 1 pg/mL.

### 2.5. Immunomagnetic Beads Remove Food Matrix Interference

**Figure 5:**
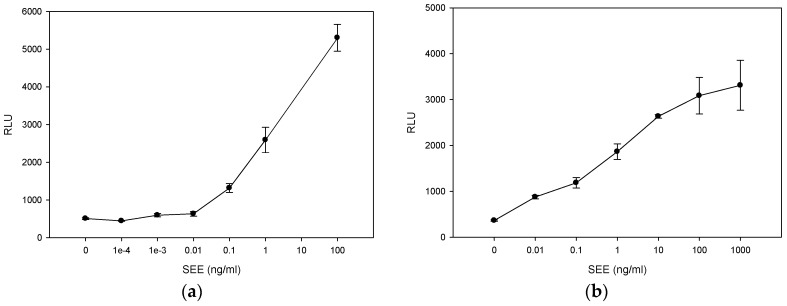
Antibody affinity influence assay sensitivity. Milk (**a**) or PBS (**b**) was spiked with increasing concentrations of SEE and incubated for 16 h with immunomagnetic beads that can remove interference from food matrix. The toxin was disassociated from beads and incubated for 5 h with the Jurkat reporter cells. The luciferase enzyme activity was determined. The photon emission was detected by luminometer. Error bars represent standard errors.

As shown in Figure 4b, food matrices are complex and often contain compounds that can interfere with the assay and increase the noise to signal ratio. To remove food matrix interference, we employed immunomagnetic beads coated with anti-SEE antibody to specifically isolate the various concentrations of SEE from spiked milk (Figure 5a) or PBS (Figure 5b). After washing three times, the toxin was eluted from the beads and was added to the cells and incubated for 5 h. As shown in Figure 5, the toxin that was eluted from the beads induced luciferase activity in a dose-dependent manner. In addition the immunomagnetic separation significantly decreased the light-emission background by 21 fold from 10,655 ± 851 RLU (Figure 4b) to 510 ± 24 RLU (Figure 5a). Nonetheless, this light-emission background level is still higher than that in PBS 363 ± 14 RLU (Figure 5b), presumably due to residual components of milk that persist despite extensive washing. In addition, due to limiting antibody binding affinity for SEE, the recovery and the signal was low (10 pg/mL).

### 2.6. Neutralizing Effect of SEE Antibody

**Figure 6:**
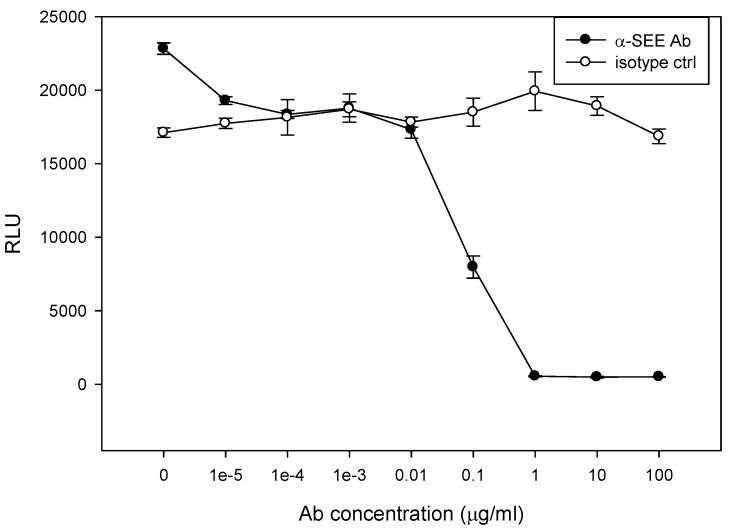
Examination of antibody’s ability to block SEE activity. Jurkat reporter cells (10,000 cells/well) were plated with Raji cells followed by addition of various concentrations of anti-SEE antibody or isotype control. The cells were stimulated in a 96-well microtiter plate with 1 ng/mL of SEE for 5 h. The photon emission was detected by luminometer. Error bars represent standard errors.

Therapeutic modalities based on antibodies which neutralize biological effects of SEs, have shown clinical success [29,30]. Performance of candidate antibody for immunotherapy against SEE depends not only upon the presence of the antibody and its binding capability, but also the ability to neutralize the biological activity of SEE. To determine whether the assay can be used to examine an antibody’s ability to block SEE activity, various concentrations of an anti-SEE antibody and isotype control, ranging from 10 μg /mL to 100 pg/mL, were incubated with SEE, Jurkat reporter cells, and Raji cells in microtiter plate. As shown in Figure 6, the data is reflecting a reverse sigmoidal curve; at low antibody concentration, the curve is relatively smooth at concentration of 10 ng/mL, and then the curve becomes steep at the equivalence point when a small increase in antibody concentration results in a large inhibition change. The isotype control antibody that we used to measure the level of non-specific background signal caused by IgG primary antibodies, on the other hand, did not neutralize the biological activity of SEE.

## 3. Discussion

For many years, we have had a strong desire to replace the *in vivo* bioassays for detection of SEs that measure emetic activity in kitten and monkey. These activity tests involve intravenous administration of toxin into kittens or administrating a 25–100 mL toxin containing food sample by gavage into the rhesus monkey’s stomach and observing vomiting reaction. On administration of 10 mg SEs, vomiting occurs only with 50% of the animals [16,17,18]. This expensive procedure has low sensitivity and poor reproducibility, requires many animals, is impractical to test on a large number of samples, and raises ethical concerns with regard to the use of experimental animals.

During our search for a replacement for the above *in vivo* assay, we discovered and demonstrated that a subtype of mouse naïve CD4^+^ T cells expresses MHC class II on their cell surface, and that these CD4^+^ T cells can perform the roles of both antigen presenting cells and T cells [7]. We tried to use this previously undescribed population of mouse naïve CD4^+^ T cells for developing activity assays for detection of SEs. However, we were unable to maintain these cells for more than 13 days. During this time the proliferation rate of this cell increased 40-fold.

In the present study, we used a Jurkat T cell reporter cell line in combination with Raji cells that perform the role of APC to measure the biological activities of SEE. In this study, we utilized the superantigenic activity of SEE by adding nonradioactive analog of thymidine that incorporates into newly synthesized mouse splenocyte DNA and demonstrated that the effect of SEE on spleen cell proliferation was similar to the effects of SEA. This proliferation bioassay is based on SEE superantigen activity and can detect 10 ng/mL of SEE, which is 100 times lower than the SEA test used with human peripheral blood mononuclear cells with a sensitivity of 0.1 ng/mL [31] and 20 times more sensitive than when radioisotope ^3^H thymidine was used [32]. Even though this *ex vivo* bioassay dramatically reduces the number of animals used (one mouse spleen can be used for 500 tests), this assay still requires the sacrifice of live animals. Ideally, an alternate assay would not require the harvesting of any tissues from living animals. Our previous work with mouse splenocytes showed that in only 6 h after stimulation, SEA induces intracellular signals that lead to differential expression of CD4^+^ T cell surface receptor CD154 [21] and secretion of tumor necrosis factor (TNF) in a time and dose dependent manner [9]. Kaminuma *et al.* show that Nuclear Factor of Activated T-cell (NFAT) is expressed soon after stimulation and that this protein mediates the expression of TNF [33] and T cell surface receptor CD154 [22].

It was shown that SEs are recognized and interact with particular sequences within the variable region of the β chain (V-β8) of the T cell receptor (TCR) in Jurkat cells [23,24]. This fact, combined with our previous work with TNF and CD154, led us to explore the utility of a genetically engineered T cell line expressing the luciferase reporter gene under the control of NFAT response element (NFAT-RE). This study reports the successful use of this genetically engineered T cell line in Jurkat, combined with a B-cell line that presents the SEE-MHC class II complex to the engineered T cell line. This assay is fast, simple, and inexpensive alternative to the *in vivo* bioassay.

Previously, we showed that, 6 h after SEA exposure, intracellular signals were triggered leading to differential expression of CD4^+^ T cell surface receptor CD154. What is shown in this present study is that five hours after exposure to SEE, this mixed culture produces luciferase enzyme. In the presence of substrate, the enzyme generated light levels proportional to SEE concentration with linear correlation *R*^2^ = 0.99. The limit of detection of biologically active SEE is 1 fg/mL which is 100,000 times more sensitive than the spleen cell proliferation bioassay, a million times more sensitive than a typical ELISA assay at a detection limit of 1 ng/mL, and a billon times more sensitive than the monkey and kitten bioassay in which vomiting occurs on administration of 10 mg. Both SEE and SEA toxins require zinc for binding to MHC class II [34], and both have a segment with the HEXXH zinc-binding motif of metalloproteases [25]. Both belong to the same SEs group based on amino acid sequence homology [26,27]. Nonetheless, SEA does not respond to the Jurkat reporter cell line assay, even though both SEA and SEE trigger proliferation in the splenocyte assay as shown in Figure 1. It is possible that the observed difference in sensitivity of this assay to SEA *versus* SEE is a product of the differences in specificities of the SEs to different region of the β chain of the T cell receptor (TCR) in Jurkat cells. It has been shown, for example, that each different SE toxin induces the expansion of different V β subsets in human T lymphocytes [35]. Particularly noteworthy is that SEE induces Vβ 8 while SEA induces Vβs 5.2, 5.3, 7.2, 9, 16, 18 and 22.

In addition, our results show that SEE stability is dependent on the medium in which the toxin is present. SEE in PBS is more readily inactivated than in milk. This suggests that SEE is more thermostable in milk and that milk has a protective effect on SEE. Pasteurization, which destroys spoilage microorganisms and reduces the number of viable pathogens, such as *S. aureus,* slightly reduces SEE activity although SEE will still be present after a longer and higher heat treatment. This demonstrates a need to prevent early contamination of *S. aureus* in milk. Once the toxin has formed, it is impractical to eliminate it from milk by thermal treatment without compromising the quality of the milk.

## 4. Materials and Methods

### 4.1. Toxin and Cell Lines

SEE, anti-SEE and isotype control antibody were obtained from Toxin Technology (Sarasota, FL, USA). The toxin concentrations (100 μg) were quantified by Toxin Technology, INC. T cell leukemia Jurkat cell line stably expressing luciferase reporter driven by NFAT response element, called GloResponse™ NFAT-RE-luc2 Jurkat Cells (also called Jurkat reporter cell line) was a generous gift from Promega. The Jurkat reporter cell line was cultured in RPMI 1640 medium (GIBCO, Carlsbad, CA, USA, #22400) supplemented with 10% fetal calf serum, 1% MEM Non Essential Amino Acids (Invitrogen, Carlsbad, CA, USA, #11140), Sodium Pyruvate 100 nM (Invitrogen, #11360), and Hygromycin B 200 μg/mL (Invitrogen, #10687-010). Burkitt’s lymphoma Raji B cell line (ATCC number CCL-86) was purchased from the American Type Culture Collection (ATCC, Rockville, MD, USA). The Raji B cell line was cultured in RPMI 1640 medium (Gibco, #22400) supplemented with 10% fetal calf serum, 1% MEM Non Essential Amino Acids (Invitrogen, #11140), Sodium Pyruvate 100 nM (Invitrogen, #11360), and 100 units/mL penicillin, 100 μg/mL streptomycin (GIBCO, #15140-122). Cells were maintained in a humidified incubator at 37 °C with 5% CO_2_. We partially inactivated the SEE by heating various diluted SEE samples in 1.5 mL Eppendorf tubes at 99 °C on a heat block for 5 min. Nonfat dry milk from Nestlé Carnation (Vevey, Switzerland) was dissolved in distilled water to form 5.0% solutions.

### 4.2. Ethics Statement

All procedures with animals were carried out according to institutional guidelines for husbandry approved by the Institutional Animal Care and Use Committee of the U.S. Department of Agriculture, Western Regional Research Center (USDA IACUC). This specific procedure and protocol was reviewed and approved by the USDA IACUC (Protocol #13-1, 5 January 2016). Mice were euthanized using rapid cervical dislocation to minimize suffering.

### 4.3. Splenocyte Isolation

Spleens were aseptically removed from C57BL/6 female mice and were disrupted by needle and syringe using Russ-10 cell culture medium. The cells were pelleted by centrifugation at 200× *g* for 10 min. at 4 °C. Red blood cells were removed by resuspending the pellet in red cell lysis buffer followed by a final centrifugation and resuspension in Russ-10 medium. A viable cell count was performed using a hemocytometer and trypan blue. Russ-10 cell culture medium was prepared by combining 450 mL of RPMI 1640 medium lacking l-glutamine (Gibco/Life Technologies, Carlsbad, CA, USA), 50 mL fetal bovine serum (Hyclone, Logan, UT, USA), 5 mL 200 mM glutamine (Gibco), 5 mL antibiotic-antimycotic (Gibco), 5 mL nonessential amino acid mix (Gibco), 5 mL sodium pyruvate (Gibco), and 0.25 mL of 100 mM β-mercaptoethanol (Sigma, St. Louis, MO, USA). Red cell lysis buffer comprised a solution containing 0.15 M NH_4_Cl, 10 mM KHCO_3_ and 0.1 mM Na_2_EDTA.

### 4.4. SEE Magnetic Bead Preparation

Dynabeads M-280 tosylactivated (Invitrogen, Carlsbad, CA, USA) were prepared by washing twice with 600 μL of pH 9.5 sodium borate (0.1 M) buffer per 100 μL beads and finally diluting to 2 × 10^9^ beads mL^−1^ in the same buffer. Purified anti-SEE antibody was covalently bound to the beads by incubation of 30 μg of the antibody with 50 μL of beads (*i.e.*, 1 × 10^8^ beads) for 24 h at 37 °C with slow shaking. To remove unbound antibody, the beads were twice washed in 1 mL PBS-BSA buffer (phosphate buffered saline (PBS), pH 7.4, containing 0.1% bovine serum albumin (BSA)) for 5 min at 4 °C, once washed in TRIS-BSA buffer (0.2 M Tris-HCl, pH 8.5, containing 0.1% BSA) for 4 h at 37 °C and washed once more with PBS-BSA buffer for 5 min at 4 °C. The beads were finally resuspended in 50 μL TRIS-BSA buffer.

### 4.5. Sample Binding and Disassociation of SEE from Beads

Samples (4 mL) of SEE spiked milk or PBS were incubated with 15 μL of the anti-SEE coated immunomagnetic beads for 24 h at 4 °C on a tilting platform. Sample tubes were placed on a magnet for 2 min to collect the beads which were then washed twice with PBS-BSA buffer. SEE toxin was eluted from the beads using 7.5 mL of 100 mM glycine-HCl (pH 2.5) followed by neutralization with 7.5 mL of 2× Tris-buffered saline (TBS) (100 mM Tris, 300mM NaCl, adjusted to pH 8.3).

### 4.6. Ex Vivo Assay for SEE Detection

Splenocytes were treated with SEE at selected concentrations in 96-well plates containing 200,000 cells per well in 200 μL Russ-10 medium. Plates were incubated 48 h at 37 °C in a 5% CO_2_ incubator. Cell proliferation was measured by the incorporation of bromodeoxyuridine (5-bromo-2-deoxyuridine, BrdU) into DNA of dividing cells. BrdU was added 4 h before fixation as described by the manufacturer (Calbiochem, San Diego, CA, USA) followed by spectroscopic measurement of OD at 620 nm and 450 nm.

### 4.7. In Vitro Assay for SEE Detection

Jurkat Reporter cells were plated in a 96-well black microplate with clear bottom, 50 μL (100,000 cells)/well, without Hygromycin B. Raji cells endogenously expressing MHC II were then added at 25 μL (50,000 cells)/well before 4X SEE (4 μg/mL) was added (25 μL/well) to the above mixed culture for a final concentration of 1 μg/mL SEE. That is, the SEE was 4 times more concentrated than the final concentration. The plates incubated for 5 h at 37 °C. At the end of the incubation, the assay plate was removed from the incubator and allowed to equilibrate to ambient temperature (10–15 min). Bio-Glo reagent (100 μL/well) was added to each well and incubated for 5–10 min at room temp. The luciferase enzyme activity was determined according the manufacturer’s instructions (Promega, Madison, WI, USA). Luminescence was measured using a luminescence plate reader.

### 4.8. Neutralizing Effect of SEE Antibody

To determine whether neutralization assay can be used to examine an antibody’s ability to neutralize SEE’s biological activity, various concentrations of an anti-SEE antibody or isotype control IgG, ranging from 10 μg /mL to 100 pg/mL, were incubated for 5 h at 37 °C with 1 ng/mL of SEE, Jurkat reporter cells, and Raji cells on a microtiter plate. At the end of the incubation, the assay plate was removed from the incubator and allowed to equilibrate to ambient temperature (10–15 min). Bio-Glo reagent (100 μL/well) was added to each well and incubated for 5–10 min at room temp. The luciferase enzyme activity was determined according the manufacturer’s instructions (Promega, Madison, WI, USA). Luminescence was measured using a luminescence plate reader.

## Abbreviations

The following abbreviations are used in this manuscript:

SEs	Staphylococcal enterotoxins
SEE	Staphylococcal enterotoxin type E
SEA	Staphylococcal enterotoxin type A
MHC II	Major histocompatibility complex class II
TCR	T-Cell receptor
NFAT	Nuclear factor of activated T-Cell
NFAT-RE	Nuclear factor of activated T-Cell response element
APC	Antigen presenting cell
TNF	Tumor necrosis factor
IFN	Interferon
MS	Mass spectrometry
ELISA	Enzyme-linked immunosorbent assay
PBS	Phosphate buffered saline
BSA	Bovine serum albumin
TBS	Tris buffered saline
BrdU	5-bromo-2-deoxyuridine
